# Jak2-Independent Activation of Stat3 by Intracellular Angiotensin II in Human Mesangial Cells

**DOI:** 10.1155/2011/257862

**Published:** 2011-09-12

**Authors:** Rekha Singh

**Affiliations:** Hines VA Medical Center, Hines, IL 60141, USA

## Abstract

Ang II is shown to mediate the stimulatory effect of high glucose on TGF-b1 and extracellular matrix proteins in glomerular mesangial cells. Also inhibition of Ang II formation in cell media (extracellular) and lysates (intracellular) blocks high-glucose effects on TGF-b1 and matrix more effectively compared to inhibition of extracellular Ang II alone. To investigate whether intracellular Ang II can stimulate TGF-b1 and matrix independent of extracellular Ang II, cultured human mesangial cells were transfected with Ang II to increase intracellular Ang II levels and its effects on TGF-b1 and matrix proteins were determined. Prior to transfection, cells were treated with candesartan to block extracellular Ang II-induced responses via cell membrane AT1 receptors. Transfection of cells with Ang II resulted in increased levels of intracellular Ang II which was accompanied by increased production of TGF-b1, collagen IV, fibronectin, and cell proliferation as well. On further examination, intracellular Ang II was found to activate Stat3 transcription factor including increased Stat3 protein expression, tyrosine 705 phosphorylation, and DNA-binding activity. Treatment with AG-490, an inhibitor of Jak2, did not block intracellular Ang II-induced Stat3 phosphorylation at tyrosine 705 residue indicating a Jak2-independent mechanism used by intracellular Ang II for Stat3 phosphorylation. In contrast, extracellular Ang II-induced tyrosine 705 phosphorylation of Stat3 was inhibited by AG-490 confirming the presence of a Jak2-dependent pathway. These findings suggest that intracellular Ang II increases TGF-b1 and matrix in human mesangial cells and also activates Stat3 transcription factor without involvement of the extracellular Ang II signaling pathway.

## 1. Introduction

Kidney damage is one of the long-term complications of diabetes (diabetic nephropathy) which is characterized by excessive production of extracellular matrix by glomerular mesangial cells. Angiotensin II (Ang II), a growth-promoting hormone derived from the renin angiotensin system (RAS), is suggested to play an important role in transmitting high glucose effects on mesangial matrix [1]. Similar to glucose, Ang II increases matrix synthesis [2] and decreases matrix degradation [3] leading to matrix accumulation in mesangial cells. Both glucose and Ang II appear to involve transforming growth factor-beta 1 (TGF-b1) for their actions on mesangial matrix. Previous studies have reported that high glucose causes increase in TGF-b1 mRNA expression and protein in mesangial cells [4, 5]. Also, Ang II is found to stimulate TGF-b1 secretion in rat mesangial cells as demonstrated by our previous studies [3]. Because these actions of Ang II are similar to those of glucose, it is likely that Ang II may act as a downstream mediator of high-glucose effects on TGF-b1 and matrix in mesangial cells. 

It is now well established that high-glucose milieu in diabetes causes activation of the RAS, particularly Ang II [1]. Treatment with angiotensin-converting enzyme (ACE) inhibitors and angiotensin receptor blockers (ARBs) has proven beneficial in delaying the progression of renal damage in type 1 and type 2 diabetic patients [6–8] suggesting activation of the RAS due to hyperglycemia. An increased renal vasodilator response to ACE inhibition or Ang II blockade in diabetic patients [9] has been interpreted as evidence that the intrarenal RAS is activated in diabetes. In streptozotocin- (STZ-) induced rat model of diabetes (type 1), we found increased levels of Ang II and its precursor, angiotensinogen (Agt) in glomerular extracts indicating activation of the glomerular RAS [10]. Also in type 2 diabetic rats, blockade of Ang II activity by ACE inhibitors and ARBs ameliorated progression of proteinuria and preserved glomerular structure further supporting RAS activation in diabetes [11]. 

Previous studies from our laboratory have consistently shown that high glucose activates Ang II production in mesangial cells [3, 12, 13] primarily by increasing synthesis of Agt, the precursor of Ang II [12]. In addition, exposure of mesangial cells to high glucose resulted in increased levels of Ang II in the cell lysates (intracellular) which were noticeably higher compared to extracellular Ang II levels found in the cell media [14, 15]. Further, our recent studies showed that inhibition of extracellular Ang II formation resulted in a partial block of high-glucose-induced increase in TGF-b1 and matrix, whereas suppression of both intracellular and extracellular Ang II formation by Agt knockdown produced a greater inhibition of TGF-b1 and matrix [15]. These findings led us to hypothesize that intracellular Ang II may contribute to the overall increase in TGF-b1 and mesangial matrix proteins under high-glucose condition. Therefore, the present study was designed to investigate whether intracellular Ang II can independently affect TGF-b1 and matrix in mesangial cells without involvement of the extracellular Ang II signaling pathway. Cultured human mesangial cells were transfected with Ang II to increase intracellular Ang II levels whereas candesartan was used to block activation of extracellular Ang II signaling via the cell membrane AT1 receptors. The findings of the present study suggest that intracellular Ang II can increase TGF-b1 and mesangial matrix and also activates Stat3 transcription factor independent of the extracellular Ang II signaling pathway.

## 2. Methods

### 2.1. Chemicals

Angiotensin II was purchased from Sigma Chemicals (St. Louis, Mo) and angiotensin II conjugated with fluorescein from Invitrogen (Carlsbad, CA). AG-490 and Jak inhibitor I were obtained from Calbiochem (EMD Chemicals Inc., Gibbstown, NJ). SDS, acrylamide/Bis, nitrocellulose membrane, Tween-20, ammonium persulphate, TEMED, and protein assay reagents were purchased from Bio-Rad laboratories (Hercules, CA) and other reagents from Sigma Chemicals (St. Louis, MO). Antibodies to total Stat3, *β*-actin, and goat anti-rabbit IgG conjugated with horseradish peroxidase (HRP) were obtained from Cell Signaling Technology (Danvers, MA) and anti-Jak2 antibody from CHEMICON (EMD Millipore, Danvers, MA). The protein molecular weight marker was obtained from Amersham (GE Healthcare, Piscataway, NJ) and the chemiluminescence detection kit from Pierce (Thermo Fisher Scientific, Rockford, IL). Candesartan was obtained from AstraZeneca Pharmaceuticals (Wilmington, DE).

### 2.2. Human Mesangial Cell Culture

Primary normal human mesangial cells (HMCs) were obtained from ScienCell (CA) at passage 1 and maintained in mesangial cell growth media (MsGM) (Lonza, MD) containing 5% fetal bovine serum and 1 *μ*g/mL gentamicin at 37°C in 5% CO_2_ and 95% air [15]. Cells were subcultured at 70–80% confluence, and experiments were performed on cells between 2 and 5 passages.

### 2.3. Transfection of Cells with Ang II

To study the role of intracellular Ang II specifically, intracellular levels of Ang II were increased using a protein transfection reagent (Proteojuice, Novagen, WI). Briefly, Ang II was mixed with proteojuice as per instructions from the supplier (Novagen) and incubated for 20 min at room temperature followed by 1 : 10 dilution with MsGM free of serum and supplements. Mesangial cells were then incubated with this media for 20 minutes to 24 h depending upon the experimental protocol. To inhibit binding of any free Ang II present in the proteojuice mixture to cell membrane AT1 receptors, cells were pretreated with candesartan to block AT1 receptors. At termination of experiments, cell media were collected and cells were used for preparation of either total cell lysates (in RIPA buffer) or cytosol and nuclear fractions (Active Motif, CA). Samples were stored at −70°C until analyzed.

### 2.4. Measurement of Ang II Levels by ELISA

Ang II levels in cell media (extracellular) and lysates (intracellular) were measured by a competitive inhibition ELISA (Peninsula-Bachem, Belmont, CA) as described previously by us [14]. Briefly, standards or samples along with anti-Ang II antibody and biotinylated Ang II peptide were incubated in a 96-well plate for 2 h followed by incubation with streptavidin-conjugated horseradish peroxidase for 1 h at room temperature. The final reaction in the well was developed with 3,3′,5,5′-tetramethyl benzidine (TMB) substrate, terminated with 2N HCl, and read at 450 nm using an ELISA reader. Ang II levels in the samples were calculated from an Ang II standard curve run with each assay.

### 2.5. Measurement of Matrix Proteins and Cell Proliferation

Cell media were dialyzed, lyophilized, and reconstituted at a known protein concentration. TGF-b1 levels were measured by a sandwich ELISA which employs a primary capture antibody and the avidin-biotin peroxidase detection system (R&D Systems, Minneapolis, MN) [3]. Collagen IV and fibronectin levels in cell media were measured by ELISA using commercially available kits from Exocell (Philadelphia, PA) and CHEMICON (EMD Millipore Danvers, MA), respectively. For determination of cell proliferation, mesangial cells were seeded in 96-well plates 24–48 prior to the assay. Cells were transfected with Ang II using proteojuice and incubated at 37°C in 5% CO_2_ and 95% air for 48 h after which proliferation of cells was measured using a colorimetric method (Roche Applied Sciences, IN).

### 2.6. Study of Jak2/Stat3 Pathway

#### 2.6.1. Protein Expression of Jak2 and Stat3

Total cells lysates from mesangial cells treated with exogenous Ang II or transfected with Ang II were prepared in RIPA buffer (Santa Cruz Biotechnology, CA) and analyzed for protein expression of Jak2 and Stat3 by Western blotting. Samples were electrophoresed on 8–10% acrylamide gel and proteins transferred to nitrocellulose membrane. Incubation with anti-Jak2 or anti-Stat3 antibodies was carried out overnight at 4°C followed by washings and incubation with a HRP-conjugated secondary antibody. The same membranes were stripped, and protein expression for *β*-actin (protein loading control) was determined. Protein bands were detected using chemiluminescence substrate (Pierce-Thermo Scientific, Rockford, IL) and analyzed by image analysis (Image J Software, National Institute of Health, Bethesda, MD). Results are expressed as the ratio of Jak2/*β*-actin or Stat3/*β*-actin. 

#### 2.6.2. Phosphorylation of Stat3

The phosphorylation of Stat3 was determined using a cell-based assay (SABiosciences, Frederick, MD). Briefly, human mesangial cells were seeded into 96-well cell culture plates 24–48 hr prior to the assay. Cells were divided into two sets and treated with exogenous Ang II or transfected with Ang II for 20 minutes after which media were removed and cells were fixed with 4% formaldehyde/1x phosphate buffered saline (PBS) buffer. After washes and blocking, one set of cells was incubated for 1 h at room temperature with phospho-Stat3 serine 727 or phospho-Stat3 tyrosine 705 antibodies to measure phosphorylated Stat3, and the other set of cells was incubated with a pan-Stat3 antibody to measure total Stat3. This was followed by incubation with a HRP-conjugated secondary antibody for 1 h at room temperature. The final reaction was developed with TMB and absorbance read at 450 nm using an ELISA plate reader. In each well, the antibody reaction was normalized to the relative cell number which was determined using a cell staining kit (SABiosciences). Results are expressed as the ratio of phospho-Stat3/total Stat3.

#### 2.6.3. DNA Binding Activity of Stat3

Nuclear extracts from mesangial cells were prepared and used for determination of Stat3-DNA binding activity (Clontech Laboratories, Inc., CA). In brief, nuclear extract samples were incubated in a 96-well plate coated with oligonucleotides containing the consensus DNA binding sequences for Stat3 transcription factor. Stat3 present in the sample recognized and bound to the specific consensus DNA sequence and the resulting DNA-Stat3 complex was detected by incubating the samples with a primary anti-Stat3 antibody followed by secondary incubation with an HRP-conjugated antibody. The final reaction was developed with TMB and read at 450 nm in an ELISA plate reader. The absorbance readings (OD_450_) represented binding activity of Stat3 transcription factor.

### 2.7. Statistical Analysis

Data were analyzed by Student's *t*-test and analysis of variance (ANOVA) (Instat, Graph-Pad, San Diego, CA) followed by posttest comparisons between groups. A *P* < 0.05 was considered significant. Values are expressed as mean ± SEM, and “*n*” denotes number of experiments in each group.

## 3. Results

### 3.1. Transfection of Human Mesangial Cells with Ang II

**Figure 1:**
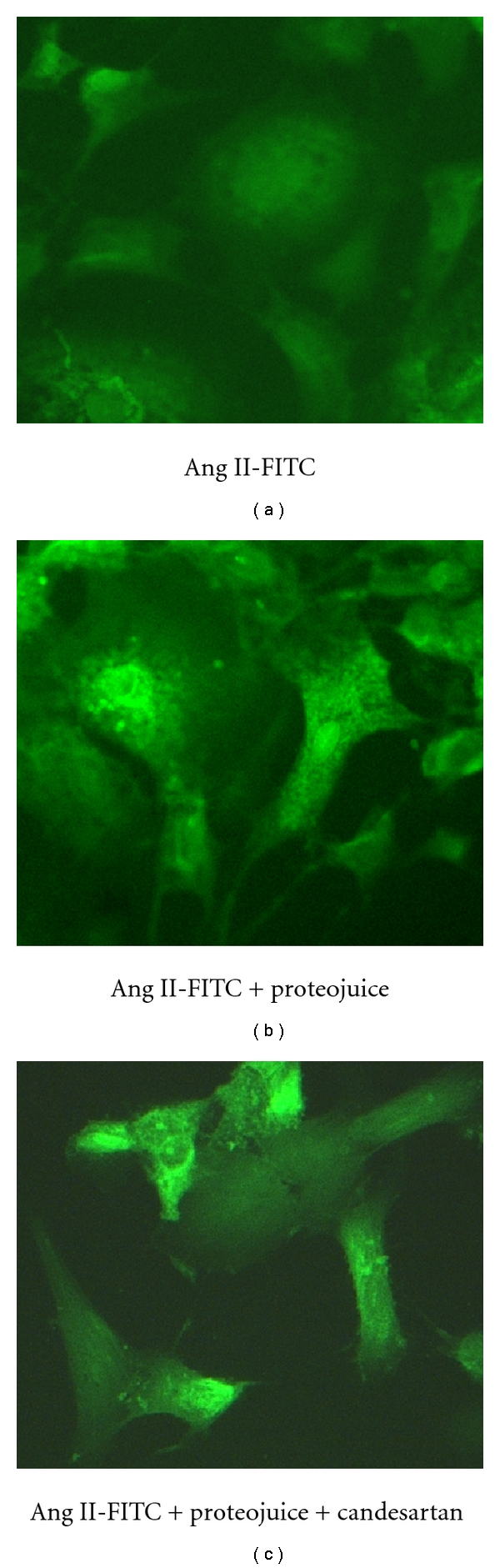
*Transfection of human mesangial cells with Ang II using proteojuice.* Human mesangial cells incubated for 30 min with a mixture of proteojuice and Ang II-FITC showed green fluorescence (b) compared to cells that were incubated with Ang II-FITC alone (a). In addition, cells incubated with proteojuice + Ang II-FITC and 100 *μ*M candesartan also showed green fluorescence (c) suggesting that treatment with Ang II receptor blocker does not interfere with intracellular delivery of Ang II by proteojuice.

First, the feasibility of transfecting primary human mesangial cells with Ang II to increase intracellular Ang II levels using proteojuice (Novagen, WI) was examined. Cells cultured in Labtek chamber slides were incubated with Ang II labeled with fluorescein (Ang II-FITC) mixed with proteojuice for 30 min and examined under epifluorescence microscope (Carl Zeiss MicroImaging Inc., NY).  Figure 1 represents a sample picture from one such experiment. Cells transfected with Ang II-FITC showed presence of green fluorescence (b) compared to nontransfected cells (a). Also, cells pretreated with 100 *μ*M candesartan (an Ang II receptor blocker) followed by transfection with Ang II-FITC showed green fluorescence (c) similar to that observed in transfected cells without candesartan treatment (b). These observations suggested that transfection of Ang II using proteojuice could deliver Ang II intracellularly and that Ang II delivery by this method is not affected by treatment with AT1 receptor blocker.

To study specific effects of intracellular Ang II on mesangial cell functions, it was important to block the extracellular Ang II signaling pathway activated by binding of any free Ang II present in the proteojuice mixture to cell membrane AT1 receptors. For this purpose, candesartan was chosen because of its physical property of binding tightly to AT1 receptor which prevents receptor activation and internalization [16]. Therefore, in all further experiments, mesangial cells were pretreated with candesartan (100 *μ*M) for 1 h and then transfected with Ang II (1 *μ*M) using proteojuice transfection reagent. Candesartan was found to have no effect on proteojuice delivery of Ang II into mesangial cells (Figure 1).

### 3.2. Ang II Delivery by Proteojuice Increases Intracellular Levels of Ang II

**Figure 2:**
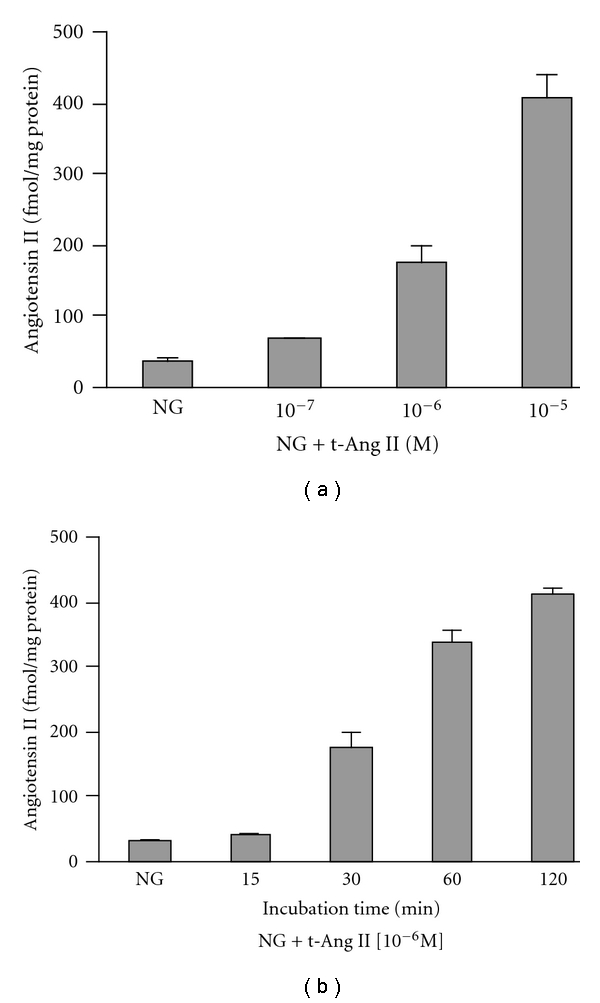
*Effect of Ang II transfection by proteojuice on intracellular Ang II levels.* (a) Human mesangial cells transfected with Ang II (NG + t-Ang II) showed a dose-dependent increase in intracellular Ang II levels with a 5-fold increase at 10^−6^ M Ang II concentration. NG represents cells incubated with 10 mM glucose alone. (b) In separate experiments, transfection of cells with 10^−6^ M Ang II raised intracellular Ang II levels in a time-dependent fashion showing a 9-fold increase after 120 min of incubation. Data are presented as mean ± SEM (*n* = 3). NG represents the mean of pooled values corresponding to the 4 time points.

To determine optimum conditions for increasing intracellular Ang II levels by the proteojuice transfection method, mesangial cells were incubated with 10 mM glucose (NG) alone or NG containing proteojuice and 10^−7^–10^−5^ M of Ang II (NG + t-Ang II) for 30 min followed by measurement of intracellular Ang II in cell lysates. Intracellular Ang II levels increased with increasing concentrations of Ang II in the proteojuice mixture showing ~1.7-fold increase with 10^−7^ M Ang II (Figure 2(a)). Also, increases of ~5-fold and ~10-fold in intracellular Ang II levels were observed with 10^−6^ M and 10^−5^ M Ang II, respectively (Figure 2(a)). 

In separate experiments, mesangial cells were incubated with NG or NG containing 10^−6^ M Ang II and proteojuice (NG + t-Ang II) mixture for 15 min-2h. A 1.5-fold increase in Ang II levels in the cell lysates (intracellular) was observed after 15 min of incubation (Figure 2(b)). Further, intracellular Ang II levels were increased by ~6-fold after 30 min, ~8-fold after 60 min, and ~9-fold after 120 min, (Figure 2(b)). These results showed a concentration- and time-dependent increase in intracellular Ang II levels in response to Ang II transfection in mesangial cells.

### 3.3. Intracellular Ang II Increases TGF-b1, Collagen IV, Fibronectin and Cell Proliferation

**Figure 3:**
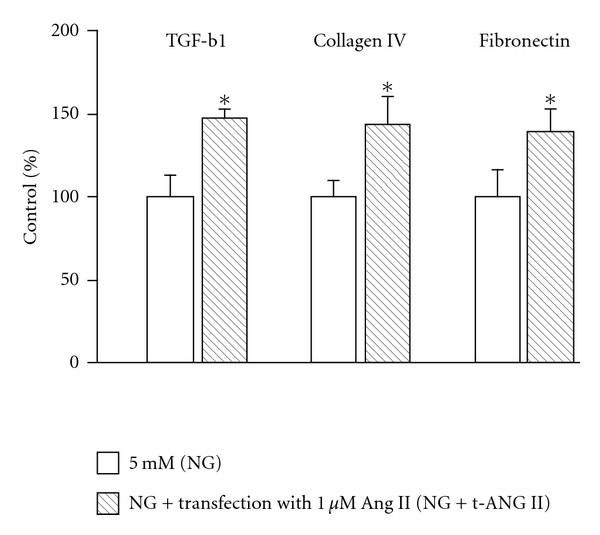
*Effect of intracellular Ang II on TGF-b1, collagen IV, and fibronectin. *Cell media from mesangial cells transfected with Ang II (NG + t-Ang II) for 24 h showed a significant increase in TGF-b1, collagen IV, and fibronectin levels compared to cells incubated in 5 mM glucose (NG) alone. Data are presented as mean ± SEM (*n* = 5). *P < 0.05 versus NG.

Next, the effects of increased intracellular Ang II levels on TGF-b1 and matrix proteins such as collagen IV and fibronectin were determined. Mesangial cells were incubated with 5 mM glucose alone (NG; control group) or NG containing a mixture of Ang II and proteojuice (NG + t-Ang II; transfection group) for 24 h, and cell media were analyzed for TGF-b1, collagen IV, and fibronectin levels. In Ang II transfected cells (NG + t-Ang II), TGF-b1 levels were significantly increased compared to control cells (NG + t-Ang II: 147 ± 6% versus NG: 100 ± 3%; Figure 3) suggesting increased secretion of TGF-b1 in response to elevated intracellular Ang II levels. In Ang II transfected cells, the increase in TGF-b1 was accompanied by increases in levels of collagen IV (NG + t-Ang II: 144 ± 18%) and fibronectin (NG + t-Ang II: 140 ± 14%) (Figure 3). 

Additionally, increased intracellular Ang II levels in Ang II-transfected cells (NG + t-Ang II) stimulated cell proliferation compared to cells incubated in NG alone (NG + t-Ang II: 138 ± 4% versus NG: 100 ± 9%, *P* < 0.05, *n* = 5). Since Ang II-transfected cells were pretreated with candesartan, these effects of intracellular Ang II appear to be mediated by intracellular signaling mechanisms different from the extracellular Ang II signaling pathway which is activated via AT1 receptors present on the cell membrane. 

### 3.4. Intracellular Ang II Signaling: Effect on Stat3 Transcription Factor

Since Stat3 transcription factor plays a key role in Ang II-mediated growth effects in mesangial cells [17], we tested the effect of intracellular Ang II on Stat3. 

#### 3.4.1. Stat3 Protein Expression

Mesangial cells were incubated in 5 mM glucose (NG; control) or NG containing 1 *μ*M Ang II mixed with proteojuice (NG + t-Ang II) for 24 h. Prior to transfection with Ang II, cells were treated with 100 *μ*M candesartan for 1 h to block cell membrane AT1 receptors. In these experiments, mesangial cells incubated with 1 *μ*M exogenous Ang II alone (NG + ex-Ang II) or in combination with 100 *μ*M candesartan (NG + C + ex-Ang II) were included as controls since exogenous Ang II is shown to activate Stat3 via AT1 receptors in these cells (17). After 24 h of treatment, experiments were terminated, and total cell lysates (RIPA buffer) were prepared. Protein expression of total Stat3 was determined by Western blotting.

**Figure 4:**
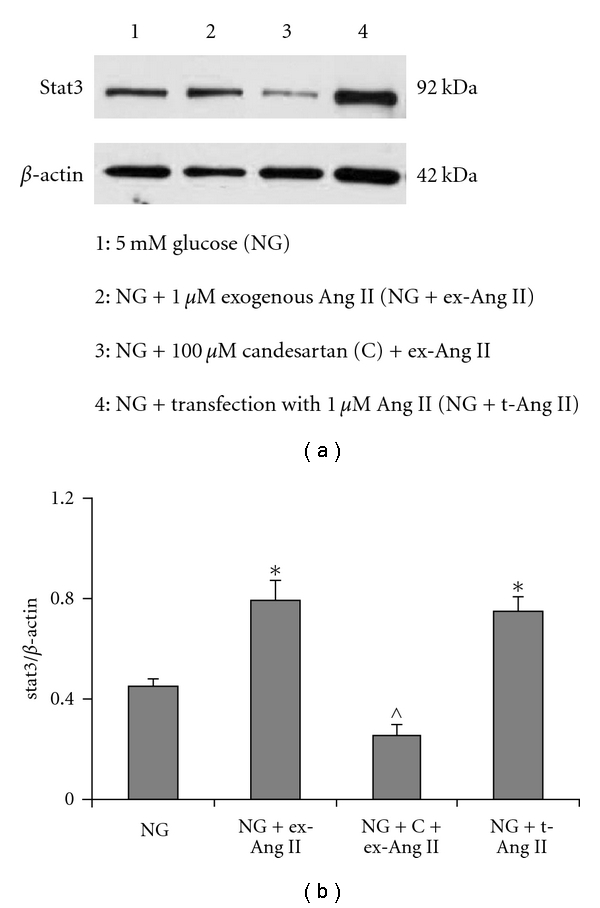
*Effect of intracellular Ang II on Stat3 protein expression*. (a) A sample picture of Western blot of total cell lysates from mesangial cells incubated with exogenous Ang II (NG + ex-Ang II) or transfected with Ang II (NG + t-Ang II) for 24 h showing Stat3 protein band (92 kDa) and b-actin (42 kDa) (loading control). (b) Densitometry analysis revealed a significant increase in Stat3 protein in cells treated with exogenous Ang II (NG + ex-Ang II) which was blocked by candesartan (C). In Ang II transfected cells (NG + t-Ang II), Stat3 protein expression increased significantly similar to that observed in cells treated with exogenous Ang II. Data are presented as mean ± SEM (*n* = 5). *P < 0.05 versus NG; $\hat{\;}P<0.05$ versus NG + ex-Ang II.

As shown in Figure 4(a), mesangial cells treated with exogenous Ang II or transfected with Ang II showed increased protein expression of Stat3 transcription factor. Densitometry analysis of Western blots revealed a significant increase in Stat3 protein expression in cells treated with exogenous Ang II which was blocked by candesartan (Figure 4(b)). These observations are in agreement with earlier reports showing activation of Stat3 by exogenous Ang II via AT1 receptors [17]. In mesangial cells transfected with Ang II (NG + t-Ang II), a significant increase in Stat3 protein expression was observed in response to increased levels of intracellular Ang II (Figure 4(b)). Because cells in NG + t-Ang II group were treated with candesartan prior to transfection with Ang II, these results suggest that the effect of intracellular Ang II on Stat3 protein is not mediated by the AT1 receptor-linked extracellular signaling pathway. 

#### 3.4.2. Stat3 Phosphorylation

**Figure 5:**
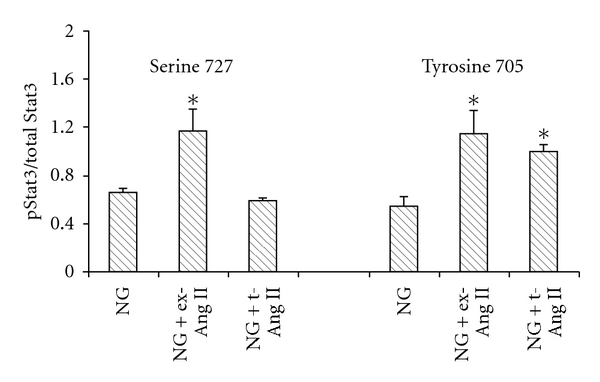
*Effect of intracellular Ang II on Stat3 phosphorylation.* Mesangial cells incubated with 5 mM glucose (NG) containing 1 *μ*M exogenous Ang II (NG + ex-Ang II) for 20 minutes showed a significant increase in both tyrosine 705 and serine 727 Stat3 phosphorylation compared to NG control. In contrast, transfection of cells with 1 *μ*M Ang II (NG + t-Ang II) for 20 minutes increased Stat3 phosphorylation only at tyrosine 705 but not at serine 727 residue. Data are presented as mean ± SEM (*n* = 3). *P < 0.05 versus NG.

Mesangial cells were cultured in 96-well plates and incubated with 5 mM glucose (NG) or NG containing 1 *μ*M Ang II mixed with proteojuice (NG + t-Ang II) or NG with 1 *μ*M exogenous Ang II (NG + ex-Ang II) for 20 minutes. At termination of the incubation period, cells were fixed and then assayed for levels of pStat3 or total Stat3 as described in Methods. Mesangial cells treated with exogenous Ang II (NG + ex-Ang II) showed significant increases in phosphorylation of Stat3 at both serine 727 (NG + ex-Ang II: 1.17 ± 0.18 versus NG: 0.66 ± 0.03) and tyrosine 705 residues (NG + ex-Ang II: 1.15 ± 0.19 versus NG: 0.54 ± 0.08) (Figure 5). In contrast, Ang II-transfected cells showed a significant increase in Stat3 phosphorylation at tyrosine 705 residue (NG + t-Ang II: 1.0 ± 0.06 versus NG: 0.54 ± 0.08) but not at serine 727 residue (NG + t-Ang II: 0.59 ± 0.2 versus NG: 0.66 ± 0.03) (Figure 5). 

#### 3.4.3. Stat3 Binding Activity

Since tyrosine 705 phosphorylation is required for Stat3 nuclear translocation and DNA binding, the effect of intracellular Ang II on Stat3 DNA binding activity was examined. Mesangial cells were incubated with 5 mM glucose (NG), NG containing 1 *μ*M exogenous, or transfected with 1 *μ*M Ang II for 24 h, and nuclear extracts were prepared and assayed for Stat3 DNA binding activity. A significant increase in Stat3 DNA binding activity was observed in mesangial cells treated with exogenous Ang II (NG + ex-Ang II) or transfected with Ang II (NG + t-Ang II) compared to NG control (NG + ex-Ang II: 128 ± 8%; NG + t-Ang II: 126 ± 3%; NG: 100 ± 11%; *n* = 4; *P* < 0.05 versus NG). Thus, these results showed that intracellular Ang II increased Stat3 phosphorylation (Tyr705) and DNA binding activity as well.

### 3.5. Role of Jak2 in Intracellular Ang II-Induced Activation of Stat3

Jak2, a cytosolic tyrosine kinase, is shown to cause activation of the latent cytoplasmic transcription factor such as Stat3 in mesangial cells [17]. For this reason, the role of Jak2 in intracellular Ang II-induced activation of Stat3 was investigated by utilizing Jak2 inhibitors such as AG-490 and Jak inhibitor I. AG-490 was chosen because in mesangial cells, it has been shown to inhibit Ang II-induced collagen IV protein synthesis [18] and high-glucose-induced increase in TGF-b1 and fibronectin synthesis along with inhibition of Stat3 tyrosine phosphorylation [19]. Jak inhibitor I is a more selective inhibitor of Jaks with much less effects on other kinases (Calbiochem EMD Chemicals Inc., NJ). 

#### 3.5.1. Effect of Jak2 Inhibition on Stat3 Protein Expression

**Effect of AG-490**

**Figure 6:**
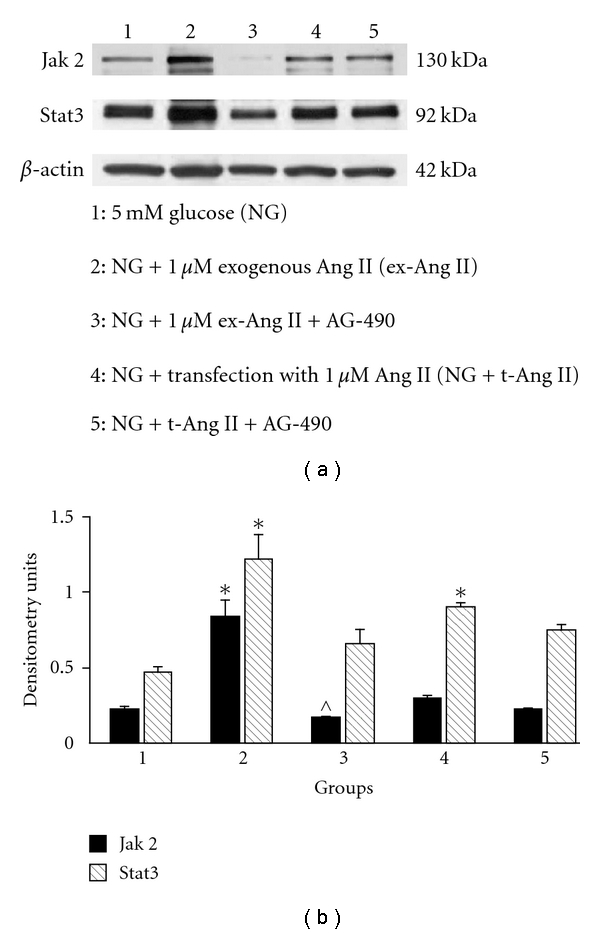
*Role of Jak2 in intracellular Ang II-induced activation of Stat3 in human mesangial cells.* (a) A sample picture of Western blot analysis showing specific protein bands for Jak2 (130 kDa), Stat3 (92 kDa), and b-actin (42 kDa) (loading control). (b) Densitometry analysis of Western blots revealed a significant increase in Jak2 and Stat3 protein expression in mesangial cells incubated with 1 *μ*M exogenous Ang II (NG + ex-Ang II) for 24 h. In contrast, transfection of cells with Ang II (NG + t-Ang II) for 24 h increased Stat3 protein expression without any effect on Jak2. Treatment with AG-490 inhibited Jak2 and Stat3 in NG + ex-Ang II group but not in NG + t-Ang II group. Data are presented as mean ± SEM (*n* = 3). *P < 0.05 versus NG; $\hat{\;}P<0.05$ versus NG + ex-Ang II.

Human mesangial cells were incubated with 5 mM glucose (NG; control) or NG containing 1 *μ*M of exogenous Ang II (NG + ex-Ang II) or NG containing 1 *μ*M Ang II mixed with proteojuice (NG + t-Ang II) for 24 h. In separate groups, cells were coincubated with exogenous Ang II or Ang II/proteojuice mixer and 10 *μ*M AG-490 for 24 h. At termination of experiments, total cell lysates were prepared and analyzed for Jak2 and Stat3 protein expression by Western blotting. As shown in Figure 6(a), exogenous Ang II increased Jak2 as well as Stat3 protein expression, whereas intracellular Ang II increased Stat3 protein without any effect on Jak2. Densitometry analysis of Western blots showed a significant increase in Stat3 protein in cells treated with exogenous Ang II or transfected with Ang II compared to NG controls (Figure 6(b)). Treatment with AG-490 inhibited exogenous Ang II-induced increase in Stat3 protein but failed to block increase in Stat3 protein expression in Ang II-transfected cells (Figure 6(b)). These findings suggest that the effect of intracellular Ang II on Stat3 may be mediated via a Jak2-independent mechanism.

**Effect of Jak Inhibitor I**

**Figure 7:**
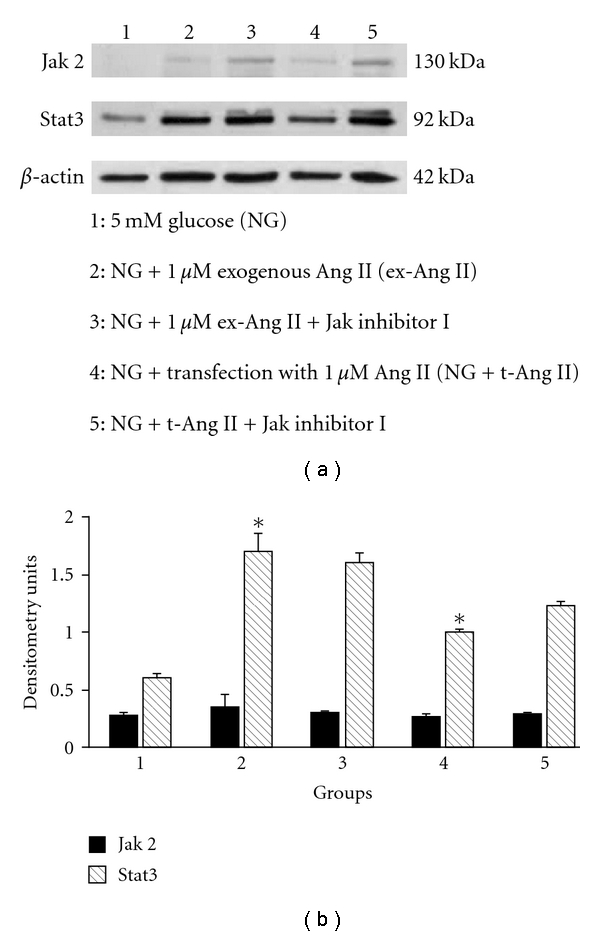
*Role of Jak2 in intracellular Ang II-induced activation of Stat3 in human mesangial cells.* (a) A sample picture of Western blot analysis showing specific protein band for Jak2 (130 kDa), Stat3 (92 kDa), and b-actin (42 kDa) (loading control). (b) Densitometry analysis of Western blots revealed a significant increase in Stat3 protein expression in mesangial cells incubated with 1*μ*M exogenous Ang II (NG + ex-Ang II) or transfected with Ang II (NG + t-Ang II) for 24 h. Treatment with Jak inhibitor I did not block increase in Stat3 protein in either NG + ex-Ang II or NG + t-Ang II group. Data are presented as mean ± SEM (*n* = 3). *P < 0.05 versus NG.

To study the effect of Jak inhibitor I on intracellular Ang II-induced increase in Stat3 protein, experiments were set up as described above for AG-490 except that 10 *μ*M Jak inhibitor I was added to the media of cells incubated with 1 *μ*M exogenous Ang II (NG + ex-Ang II or 1 *μ*M Ang II mixed with proteojuice (NG + t-Ang II). After 24 h, experiments were terminated, and total cell lysates were prepared and analyzed for Jak2 and Stat3 protein expression by Western blotting. Both treatment with exogenous Ang II or transfection with Ang II increased Stat3 protein expression in mesangial cells (Figure 7(a)). Densitometry analysis also revealed an increase in Stat3 protein in cells treated with exogenous Ang II or transfected with Ang II (Figure 7(b)). Treatment with Jak2 inhibitor I failed to inhibit increase in Stat3 protein in either exogenous Ang II-treated or Ang II-transfected cells (Figure 7(b)). Also, there was no effect of Jak inhibitor I on Jak2 protein expression in cells treated with exogenous Ang II or transfected with Ang II (Figures 7(a) and 7(b)). Since Jak inhibitor I primarily targets Jak1, it is likely that it may not have any effects on Jak2 in human mesangial cells as suggested by these results.

#### 3.5.2. Effect of Jak Inhibition on Stat3 (Tyr705) Phosphorylation

**Figure 8:**
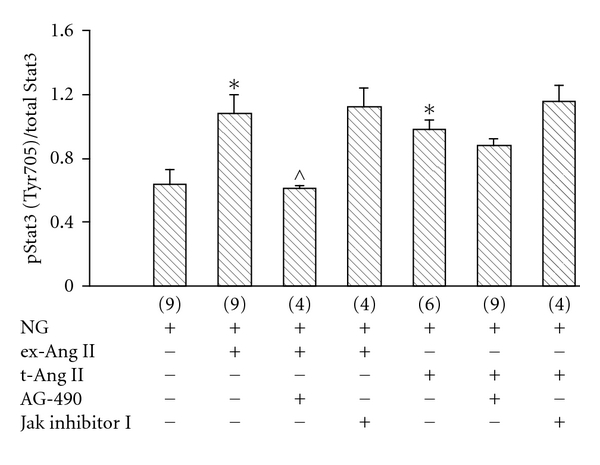
*Effect of Jak2 inhibition on intracellular Ang II-induced tyrosine 705 (Tyr705) phosphorylation of Stat3. *Incubation of mesangial cells with 1 *μ*M exogenous Ang II (ex-Ang II) for 20 minutes increased (Tyr705) Stat3 phosphorylation compared to control cells incubated in 5 mM glucose (NG), and this increase was inhibited by AG-490. In contrast, transfection of mesangial cells with Ang II for 20 minutes (NG + t-Ang II) increased Stat3 (Tyr705) phosphorylation which was not blocked by AG-490. Treatment with Jak inhibitor I had no effect on Stat3 (Tyr705) phosphorylation in cells treated with exogenous Ang II or transfected with Ang II. Data are presented as mean ± SEM of observations shown in parentheses for each group. *P < 0.05 versus NG; $\hat{\;}P<0.05$ versus NG + ex-Ang II.

In further experiments, the effect of AG-490 or Jak inhibitor I on intracellular Ang II-induced phosphorylation of Stat3 (Tyr705) was determined. Mesangial cells were incubated with 5 mM glucose (NG) or NG containing 1 *μ*M exogenous Ang II (NG + ex-Ang II) or NG containing 1 *μ*M Ang II mixed with proteojuice (NG + t-Ang II). Also, cells treated with exogenous Ang II or transfected with Ang II were incubated with 10 *μ*M of either AG-490 or Jak inhibitor I. After 20 minutes of incubation, cells were fixed and assayed for phosphorylated Stat3 (Tyr705) and total Stat3. A significant increase in Stat3 (Tyr705) phosphorylation was observed in cells exposed to exogenous Ang II (ex-Ang II) or transfected with Ang II (NG + t-Ang II) compared to cells incubated in 5 mM glucose (NG) alone (NG: 0.64 ± 0.09; NG + ex-Ang II: 1.08 ± 0.12; NG + t-Ang II: 0.98 ± 0.06) (Figure 8). Treatment with AG-490 did not inhibit intracellular Ang II-induced phosphorylation of Stat3 (Tyr705) in Ang II-transfected cells, whereas exogenous Ang II-initiated Stat3 (Tyr705) phosphorylation was significantly reduced in the presence of AG-490 (Figure 8). In contrast, there was no effect of Jak inhibitor I on Stat3 (Tyr 705) phosphorylation in either exogenous Ang II-treated or Ang II-transfected cells (Figure 8). These results suggest that intracellular Ang II may use a Jak2-independent mechanism for Stat3 phosphorylation (Tyr705) in contrast to a Jak2-dependent mechanism employed by exogenous (extracellular) Ang II. 

## 4. Discussion

The main objective of the present study was to determine whether intracellular Ang II could independently stimulate TGF-b1 and mesangial matrix without involvement of the extracellular Ang II signaling pathway. Cultured human mesangial cells were transfected with Ang II to increase intracellular Ang II levels, while the extracellular Ang II pathway was blocked by pretreatment of cells with candesartan, an Ang II receptor antagonist. Candesartan was chosen due to its physical property of tight binding to AT1 receptor which traps the receptor at the membrane [16] and prevents AT1 receptor-linked activation of the signaling pathway. Our results showed that transfection of mesangial cells with Ang II increased intracellular Ang II levels in a concentration- and time-dependent manner. Further, mesangial cells transfected with Ang II showed stimulation of TGF-b1, collagen IV, and fibronectin secretion in response to increased levels of intracellular Ang II. Also, mesangial cell proliferation was increased in transfected cells due to elevated levels of intracellular Ang II. Because these effects of intracellular Ang II were noted while cell membrane AT1 receptors were blocked by candesartan, our findings suggest that intracellular Ang II could initiate physiological responses without involving extracellular Ang II signaling pathways which are activated by the cell membrane AT1 receptors. 

 Most of the known effects of Ang II are induced by extracellular Ang II via activation of AT1 receptors present on the cell membrane [20]. The binding of Ang II to AT1 receptor initiates many signaling events including activation (phosphorylation) of Jak tyrosine kinases and Stat family of latent cytoplasmic transcription factors [17]. Ang II also stimulates formation of Stat3 homo- and hetrodimers complexes that translocate to the nucleus and bind to specific DNA motifs resulting in activation of the early growth response gene [21]. Several studies by Marrero and associates reported that the phosphorylation of Jak2 and Stat3 by Ang II is critical for Ang II-mediated growth effects such as activation of TGF-b1, synthesis of matrix proteins, and cell proliferation [17]. In the present study, an increase in Stat3 protein expression was found in mesangial cells treated with exogenous (extracellular) Ang II as well as transfected with Ang II (intracellular Ang II). In further experiments, the increased intracellular Ang II levels in Ang II-transfected cells was found to cause a significant increase in phosphorylation of Stat3 at tyrosine 705 (Tyr705) but not at serine 727 (Ser727) residue. This was in contrast to exogenous Ang II which caused phosphorylation of Stat3 at both tyrosine (Tyr705) and serine (Ser727) residues. Interestingly, Ang II (exogenous) was also found to induce tyrosine and serine phosphorylation of Stat3 in a study using other cell systems [22]. The same study showed that Ang II-induced phosphorylation of Stat3 at serine 727 is mediated by activation of extracellular regulated kinases 1 and 2 (ERK 1/2) [22]. In mesangial cells transfected with Ang II, we did not observe activation (phosphorylation) of ERK 1/2 in response to increased intracellular Ang II levels (data not shown) indicating that intracellular Ang II may not induce Stat3 phosphorylation at serine 727 residue. In Ang II-transfected cells, increased Stat3 phosphorylation (Tyr705) was accompanied by a significant increase in Stat3 DNA binding activity. Because cells transfected with Ang II were pretreated with candesartan, these findings suggest that intracellular Ang II causes tyrosine 705 phosphorylation of Stat3 independent of cell membrane AT1 receptors and promote Stat3 DNA binding activity which is important for activation of gene transcription. 

The role of Jak2 in extracellular Ang II-induced activation and translocation of Stat3 is well documented [17]. Studies in other cell systems have demonstrated that Ang II binding to AT1 receptor initiates a physical association between carboxyl terminal of AT1 receptor with Jak2, which is a critical event for activation of Jak2 kinase [23]. Indeed, in glomerular mesangial cells, exogenous (extracellular) Ang II is shown to activate Jak2 resulting in tyrosine phosphorylation and nuclear translocation of Stat3 [18]. In the present study, the role of Jak2 in intracellular Ang II-induced phosphorylation of Stat3 was investigated, and mesangial cells treated with exogenous Ang II were included as positive controls. Treatment with AG-490, an inhibitor of Jak2, was found to block Stat3 phosphorylation (Tyr705) in mesangial cells exposed to exogenous Ang II in agreement with earlier reports [18]. To our surprise, Jak inhibitor I failed to block phosphorylation of Stat3 (Tyr705) in response to treatment with exogenous Ang II. Whereas in mesangial cells, AG-490 is shown to inhibit the effect of exogenous Ang II on collagen IV synthesis [18] and high glucose on TGF-b1 synthesis and Stat3 tyrosine 705 phosphorylation [19], not much is known about the effects of Jak2 inhibitor I in these cells. Treatment with AG-490 in mesangial cells transfected with Ang II did not block phosphorylation of Stat3 (Tyr705) suggesting that a Jak2-independent mechanism may be involved in intracellular Ang II-induced tyrosine 705 phosphorylation of Stat3. Interestingly, in other cell system, Ang II-induced tyrosine 705 phosphorylation and nuclear translocation of Stat3 is also shown to be mediated by c-Src, a nonreceptor kinase [24]. However, the functional role of c-Src in intracellular Ang II-induced Stat3 phosphorylation (Tyr705) in human mesangial cells remains to be tested. 

At present, not much is known on the mechanisms by which intracellular Ang II can influence mesangial cell functions. Recently, it is proposed that intracellular Ang II is stored in endosomes and upon release into the cell cytoplasm may increase production of reactive oxygen species (ROS) by direct interaction with mitochondria [25]. Previous studies have also suggested that Ang II could exert intracellular effects by binding to its receptors present in various cytoplasmic organelles including the nucleus [26]. Indeed, intracellular Ang II is shown to cause calcium mobilization in renal proximal tubular cells [27] and cell proliferation in Chinese hamster ovary cells [28] independent of cell membrane AT1 receptors. Studies have also reported the existence of intracellular AT1 receptors in renal cortical nuclei [29] and in renal cortex and medulla of rat kidney [30]. Moreover, in isolated rat cortical nuclei, Ang II increased transcription of TGF-b1 mRNA by activation of nuclear AT1 receptors [31]; whether such mechanism operates in mesangial cells remains an open question. 

In summary, the present study showed that intracellular Ang II activates Stat3 via a Jak2-independent mechanism in contrast to extracellular Ang II-induced Stat3 activation which is mediated by Jak2. Since both pathways appear to converge on Stat3 using different routes, they could exert synergistic effects on activation of Stat3 transcription factor resulting in a greater stimulation of gene transcription of TGF-b1 and matrix proteins, especially under high-glucose condition when both intracellular and extracellular levels of Ang II are increased [14, 15]. It is noteworthy that intracellular Ang II-initiated responses were observed in the presence of candesartan, thus suggesting that ARBs are unable to block the intracellular component of Ang II signaling. This might also explain why these agents (ARBs) commonly used in clinical practice fail to completely block the progression of diabetic nephropathy. It is not known yet whether intracellular Ang II receptors are structurally identical to the cell membrane AT1 receptors or belong to a subclass of AT1 receptors which participate in the intracellular Ang II and/or nuclear signaling. There is clearly a need for further understanding of the intracellular Ang II receptors and/or signaling mechanisms for more effective control of RAS activity in diabetes and for better treatment of diabetic nephropathy.

## References

[1] Leehey DJ, Singh AK, Singh R, Cortes P, Mogensen CE (2006). Angiotensin II and its receptors in the pathogenesis of diabetic nephropathy. *The Diabetic Kidney*.

[2] Kagami S, Border WA, Miller DE, Noble NA (1994). Angiotensin II stimulates extracellular matrix protein synthesis through induction of transforming growth factor-*β* expression in rat glomerular mesangial cells. *Journal of Clinical Investigation*.

[3] Singh R, Alavi N, Singh AK, Leehey DJ (1999). Role of angiotensin II in glucose-induced inhibition of mesangial matrix degradation. *Diabetes*.

[4] Kolm V, Sauer U, Olgemoller B, Schleicher E (1996). High glucose-induced TGF-b1 regulates mesangial production of heparin sulfate proteoglycan. *American Journal of Physiology*.

[5] Ziyadeh FN, Sharma K, Ericksen M, Wolf G (1994). Stimulation of collagen gene expression and protein synthesis in murine mesangial cells by high glucose is mediated by autocrine activation of transforming growth factor-b. *Journal of Clinical Investigation*.

[6] Lewis EJ, Hunsicker LG, Bain RP, Rohde RD (1993). The effect of angiotensin-converting-enzyme inhibition on diabetic nephropathy. *New England Journal of Medicine*.

[7] Andersen S, Tarnow L, Rossing P, Hansen BV, Parving HH (2000). Renoprotective effects of angiotensin II receptor blockade in type 1 diabetic patients with diabetic nephropathy. *Kidney International*.

[8] Igarashi M, Hirata A, Kadomoto Y, Tominaga M (2006). Dual blockade of angiotensin II with enalapril and losartan reduces proteinuria in hypertensive patients with type 2 diabetes. *Endocrine Journal*.

[9] Lansang MC, Price DA, Laffel LMB (2001). Renal vascular responses to captopril and candesartan in patients with type 1 diabetes. *Kidney International*.

[10] Singh R, Singh AK, Leehey DJ (2005). A novel mechanism for angiotensin II formation in streptozotocin-diabetic rat glomeruli. *American Journal of Physiology*.

[11] Noda M, Matsuo T, Nagano-Tsuge H (2001). Involvement of angiotensin II in progression of renal injury in rats with genetic non-insulin-dependent diabetes mellitus (Wistar fatty rats). *Japanese Journal of Pharmacology*.

[12] Singh R, Singh AK, Alavi N, Leehey DJ (2003). Mechanism of increased angiotensin II formation in glomerular mesangial cells cultured in high glucose. *Journal of the American Society of Nephrology*.

[13] Leehey DJ, Isreb MA, Marcic S, Singh AK, Singh R (2005). Effect of high glucose on superoxide in human mesangial cells: role of angiotensin II. *Nephron*.

[14] Singh R, Leehey DJ (2007). Effect of ACE inhibitors on angiotensin II in rat mesangial cells cultured in high glucose. *Biochemical and Biophysical Research Communications*.

[15] Singh R, Choubey D, Chen J, Leehey DJ (2009). Inhibition of intracellular angiotensin II blocks high glucose effect on mesangial matrix. *Regulatory Peptides*.

[16] Fierensa FLP, Vanderheyden PML, Roggeman C, De Backer J, Thekkumkara TJ, Vauquelin G (2001). Tight binding of the angiotensin AT_1_ receptor antagonist. *Biochemical Pharmacology*.

[17] Marrero MB, Banes-Berceli AK, Stern DM, Eaton DC (2006). Role of the JAK/STAT signaling pathway in diabetic nephropathy. *American Journal of Physiology*.

[18] Amiri F, Shaw S, Wang X (2002). Angiotensin II activation of the JAK/STAT pathway in mesangial cells is altered by high glucose. *Kidney International*.

[19] Wang X, Shaw S, Amiri F, Eaton DC, Marrero MB (2002). Inhibition of the JAK/STAT signaling pathway prevents the high glucose-induced increase in TGF-b1 and fibronectin synthesis in mesangial cells. *Diabetes*.

[20] Ardaillou R, Chansel D, Chatziantoniou C, Dussaule J (1999). Mesangial AT_1_ receptors: expression, signaling, and regulation. *Journal of the American Society of Nephrology*.

[21] McWhinney CD, Hunt RA, Conrad KM, Dostal DE, Baker KM (1997). The type I angiotensin II receptor couples to Stat1 and Stat3 activation through Jak2 kinase in neonatal rat cardiac myocytes. *Journal of Molecular and Cellular Cardiology*.

[22] Chung J, Uchida E, Grammer TC, Blenis J (1997). Stat3 serine phosphorylation by ERK-dependent and independent pathways negatively modulates its tyrosine phosphorylation. *Molecular and Cellular Biology*.

[23] Ali MS, Sayeski PP, Dirksen LB, Hayzer DJ, Marrero MB, Bernstein KE (1997). Dependence on the motif YIPP for the physical association of Jak2 kinase with the intracellular carboxyl tail of the angiotensin II AT_1_ receptor. *Journal of Biological Chemistry*.

[24] Liang H, Venema VJ, Wang X, Ju H, Venema RC, Marrero MB (1999). Regulation of angiotensin II-induced phosphorylation of Stat3 in vascular smooth muscle cells. *Journal of Biological Chemistry*.

[25] Re RN, Cook JL (2010). The mitochondrial component of intracrine action. *American Journal of Physiology*.

[26] Re R (1999). The nature of intracrine peptide hormone action. *Hypertension*.

[27] Zhuo JL, Li XC, Garvin JL, Navar LG, Carretero OA (2006). Intracellular ANG II induces cytosolic Ca^2+^ mobilization by stimulating intracellular AT_1_ receptors in proximal tubule cells. *American Journal of Physiology*.

[28] Baker KM, Kumar R (2006). Intracellular angiotensin II induces cell proliferation independent of AT_1_ receptor. *American Journal of Physiology*.

[29] Licea H, Walters MR, Navar LG (2002). Renal nuclear angiotensin II receptors in normal and hypertensive rats. *Acta physiologica Hungarica*.

[30] Pendergrass KD, Averill DB, Ferrario CM, Diz DI, Chappell MC (2006). Differential expression of nuclear AT_1_ receptors and angiotensin II within the kidney of the male congenic mRen2.Lewis rat. *American Journal of Physiology*.

[31] Li XC, Zhuo JL (2008). Intracellular ANG II directly induces in vitro transcription of TGF-b1, MCP-1, and NHE-3 mRNAs in isolated rat renal cortical nuclei via activation of nuclear AT_1_ a receptors. *American Journal of Physiology*.

